# Correspondence on Typhoid Fever

**Published:** 1860-05

**Authors:** J. A. Brenneman

**Affiliations:** Davistown, Stephenson Co., Ill.


### CORRESPONDENCE ON TYPHOID FEVER.

Prof. Davis:

Dear Sir:—Permit me to address a few lines to you on the subject of an epidemic prevailing in this neighborhood. Ever since last September, *typhoid fever* was prevailing in a locality where heretofore disease was almost a stranger—one of the healthiest places in this western country. High rolling prairie, at the edge of a beautiful grove, always dry, and no stream near, or anything else which might cause disease. The patients are mostly young persons, sons and daughters of good and substantial farmers, who were always healthy, and who live on good and wholesome diet. Those are the persons mostly affected.

The disease last fall and through the winter was of a regular typhoid character, and was easyly controled—but few died. They generally ran from two to four weeks, and then slowly got well. I pursued the treatment which you pursued some five years ago, and with the very best success. Had some forty cases, and only three died.

But about the first of March, the disease took on another form, and about the half of the number who were attacked died in ten days after taking their bed.

The patient, before he takes his bed, complains of being chilly for several days, alternating with fever; also some headache, and aching in the bones. Bowels move four or five times a day. He now feels bad and takes his bed, and sends for a doctor. The doctor sees him; patient tells him he is not much sick—thinks he has no fever, (he looks rather pale and bilious.) But the doctor takes hold of his arm, and finds his pulse 120 to 130, and small; tells him to protrude his tongue, and he tries to protrude, but as often, perhaps, as he gets it out it goes right back, and is in a shaking motion all the time. Furred in the middle, with a heavy remittent coat; contracted at tip, and red around the edges. Bowels somewhat swollen, and perhaps a little tender to the touch; but not often that he complains of pain. The skin is dry and husky; sometimes sweats profusely, and extremities cold, and finger nails bluish; this may be the fourth or fifth day, and after that time the skin becomes hot and dry, and no sweating after that time.

There is also *subsultus tendinum*, and twiching of the muscles to a very great degree, and very restless; throws himself from one side of the bed to the other. If you ask him, well, how are you to-day? He replies sensible—well I don't think I am very bad, I feel comfortable; but is inclined to dose and sleep; is not much delirious through the day, if any, but some at night. Sleeps a few hours, and then wakes somewhat confused; takes his medicine, lays down, and perhaps doses awhile again rather comfortable; and so he goes on from day to day. The attendants think often he is getting better, but about the tenth day suddenly the breathing becomes stertorous, and he dies in a few hours without a struggle. Often about the time they are dying they are vomiting black matter, something like coffee dregs.

1 look upon this disease as not being the ordinary typhoid fever of the West. It is more malignant and more contagious. Whole families have it one after another—then it will affect the neighbors and those that watch with the patient. They are alarmed and so am I. The usual remedies will not be effectual, and nothing else that I called into requisition seems to have any special influence over the disease. Will you please tell us what to do.

Yours truly,

J. A. BRENNEMAN, M. D.

*Davistown, Stephenson Co., Ill.*

March 10th, 1860.

*Remarks.*—It is always difficult for the practising physician to form a satisfactory opinion concerning the special character of any given case or series of cases of disease from a written description. No language can convey an adequate idea of the physiognomy, the attitude, the movements, etc., of the patient; yet a just appreciation of these is often essential to the formation of a correct opinion. From the symptoms detailed in the foregoing letter, and the high ratio of mortality, it is evident that the disease described is something different from simple typhoid fever. The description reminds us of what some of the older writers called "congestive typhus." If we might venture an opinion in reference to the nature of the cases described by Dr. Brenneman, we should say that they were characterized by the co-existence of two prominent and serious pathological conditions.

The first consists in a highly congested condition of the mucous membrane of the intestines, as indicated by the appearances of the tongue, the early diarrhœa, and the subsequent tender and tympanitic state of the abdomen. The second and co-existing pathological state consists in early and profound depression of the functions of the organic or ganglionic nervous system, indicated by the paleness of the countenance, the small quick pulse, tremulous tongue, and more or less general subsultus.

The existence of the latter pathological condition not only favors the early and extensive softening of the mucous membrane of the ilium, but also greatly favors the early supervention of a *latent* pneumonic engorgement of the lungs. And we are inclined to think that it is by one or the other, or both of these lesions, that the fatal result was reached in so large a proportion of the cases. When the functions of the ganglionic nervous system are much depressed, passive engorgements often occur in important organs, and even determine a fatal result, without being indicated by any of those symptoms which usually accompany the more active congestions of the same structures.

The impaired susceptibilities of such patients very much diminish the acuteness of pain and the consciousness of local disease. From these pathological views we may deduce two leading and important indications for treatment. The one calls for the employment of such remedies as are calculated to allay the morbid sensitiveness, and arrest the tendency to disorganization of the mucous surface of the intestines. The other demands the use of such agents as tend to support the functions of the organic nervous structures, and maintain the vital affinities in all the tissues.

For accomplishing the first, we know of no remedy that proves efficient in a larger proportion of cases, than the combination of Oil of Turpentine and Tincture of Opium, given in the form of an emulsion. It is most easily administered, and least likely to offend the stomach, if prepared as follows:

℞ Ol. Terebinth, ʒ ii.

Tinct. Opii, ʒ ii.

Pulv. G. Acaccia, }

Sacchar. Alba, } aa. ʒ iii.

Rub together thoroughly, and add Aqua Mentha, ℥ ii. Shake, and give a fluid drachm every two, three, or four hours, according to the looseness of the bowels.

For accomplishing the second indication, we rely much on small doses of Quinine, and full doses of the Chlorates of Potassa and Soda. From one to two grains of Sulphate of Quinine, with eight or ten grains of the Chlorates, may be given between each dose of the emulsion. In all such cases of low grade of febrile disease, as described in the foregoing letter, in which the intestinal evacuations are thin and more frequent than natural at the commencement of the disease, we resort to the foregoing remedies early, and carefully abstain from all direct evacuants.

If the skin and conjunctiva are yellow, and the stools clay-colored, indicating an actual retention of the biliary secretion, we would give from one to two grains of Calomel in each of the first four or five doses of the emulsion.

The Quinine and Chlorates, by preserving the blood from degeneration, and increasing its capacity for absorbing oxygen in the lungs, more directly and efficiently sustains the action of the organic nervous structures, and promotes the susceptibility of all the tissues, than any of the so-called diffusable stimulants. While the Oil of Turpentine not only acts locally with the laudanum to lessen the irritability, and increase the tone of the capillaries of the intestinal mucous membrane, but it exerts a well-known sustaining influence over the whole vascular system, such as strongly counteracts passive congestions. Of course the foregoing are mere hints; and whether they are applicable in any degree to the fever prevalent in Dr. Brenneman's neighborhood, could only be determined by a careful personal examination, rationally and *physically*, of the cases as they occur.—[Ed. of the Examiner.]

